# Long-Term Outcomes in Patients with Early Stage Nodular Lymphocyte-Predominant Hodgkin’s Lymphoma Treated with Radiotherapy

**DOI:** 10.1371/journal.pone.0075336

**Published:** 2013-09-18

**Authors:** Abhishek A. Solanki, Melissa Horoschak LeMieux, Brian C.-H. Chiu, Usama Mahmood, Yasmin Hasan, Matthew Koshy

**Affiliations:** 1 Department of Radiation and Cellular Oncology, University of Chicago, Chicago, Illinois, United States of America; 2 Department of Health Studies, University of Chicago, Chicago, Illinois, United States of America; 3 Division of Radiation Oncology, University of Texas M. D. Anderson Cancer Center, Houston, Texas, United States of America; 4 Department of Radiation Oncology, University of Illinois Hospital, Chicago, Illinois, United States of America; The Ohio State University, United States of America

## Abstract

**Purpose:**

Radiation therapy (RT) is commonly used as definitive treatment for early-stage nodular lymphocyte-predominant Hodgkin’s lymphoma (NLPHL). We evaluated the cause-specific survival (CSS), overall survival (OS), and second malignancy (SM) rates in patients with early-stage NLPHL treated with RT.

**Methods and Materials:**

Patients with stage I-II NLPHL between 1988 and 2009 who underwent RT were selected from the Surveillance, Epidemiology and End Results database. Univariate analysis (UVA) for CSS and Os was performed using the Kaplan-Meier method and included age, gender, involved site, year of diagnosis, presence of B-symptoms, and extranodal involvement (ENI). Multivariable analysis (MVA) was performed using Cox Proportional Hazards modeling and included the above clinical variables. SM were classified as RT-related or non-RT-related. Freedom from SM and freedom from RT-related SM were determined using the Kaplan-Meier method.

**Results:**

The study cohort included 469 patients. Median age was 37 years. The most common involved sites were the head and neck (36%), axilla/arm (26%), and multiple lymph node regions (18%). Sixty-eight percent had stage I disease, 70% were male, 4% had ENI, and 7% had B-symptoms. Median follow-up was 6 years. Ten-year CSS and Os were 98% and 88%, respectively. On UVA, none of the covariates was associated with CSS. Increasing age (p<0.01) and female gender (p<0.01) were associated with worse Os. On MVA, older age (p<0.01), female gender (p=0.04), multiple regions of involvement (p=0.03), stage I disease (p=0.02), and presence of B-symptoms (p=0.02) were associated with worse Os. Ten-year freedom from SM and freedom from RT-related SM were 89% and 99%, respectively.

**Conclusions:**

This is the largest series to evaluate the outcomes of stage I-II NLPHL patients treated with RT and found that this patient population has an excellent long-term prognosis and a low rate of RT-related second malignancies.

## Introduction

The existence of nodular lymphocyte-predominant Hodgkin lymphoma (NLPHL), a variant of Hodgkin’s lymphoma, has been known for over 30 years [1,2]. Although the incidence of NLPHL appears to be increasing in children, it still only accounts for 5% of Hodgkin’s lymphoma, making it a difficult disease entity to study [3,4].

Because 80% of patients with NLPHL have early-stage (stage I-II) disease, the outcomes of this cohort are particularly important to understand [4,5]. Additionally, current National Comprehensive Cancer Network (NCCN) guidelines recommend using radiotherapy for the definitive therapy of most patients with early-stage NLPHL, making the outcomes with this treatment modality the most relevant [6]. Prior studies have revealed that patients with NLPHL appear to have favorable disease outcomes and survival compared to their counterparts with classical Hodgkin’s lymphoma [4,7,8,9]. However, these studies have been limited by small cohorts and mostly single institution experiences.

The objectives of this study were to determine the cause-specific survival and overall survival of patients with early-stage NLPHL treated with radiotherapy in the United States using a population-based cohort. In addition, we sought to estimate the second malignancy rate and radiotherapy-related second malignancy rate in this cohort.

## Methods and Materials

The Surveillance, Epidemiology, and End Results (SEER) Program of the National Cancer Institute covers 28% of the U.S. population and collects incidence and survival data from 18 population-based cancer registries (SEER-18). The database contains information on primary tumor site, age, gender, histologic type, stage at diagnosis, first course of treatment, follow-up, and cause of death.

### Data and study population

Institutional Review Board exemption for this study was obtained from the University of Chicago Institutional Review Board. As part of the exemption, the need for written informed consent from participants was waived. The eligible patients had histologically confirmed NLPHL and were treated with radiotherapy as the initial treatment modality. Patients with classical Hodgkin’s lymphoma were excluded from the present analysis. We restricted the analysis to patients who had been diagnosed between 1988 and 2009. Patients with extent of disease codes that corresponded to the current American Joint Committee on Cancer stage I and II were included, whereas those who presented with stage III or IV or an unknown stage were excluded. The potential date of last follow-up in our cohort was December 31, 2009.

### Covariates of Interest

Pertinent patient characteristics were described, including age at diagnosis, gender, year of diagnosis, primary tumor site, stage (I vs. II), extranodal involvement, and B-symptoms. Due to the few numbers in each anatomic site, the 4 most common sites were made separate subgroups (head and neck, axilla/arm, multiple lymph node regions, and inguinal/leg), and the rest were grouped together into an “other” subgroup. Patients with primary site of “multiple lymph node regions” were those who had involvement of multiple lymph nodes with lymphoma but it was not possible to identify the lymph node region where the lymphoma originated [10]. All patients in this group had Stage II disease. For all analyses, age was treated as an ordinal variable and categorized as ≤19, 20-44, 45-59, and ≥60). Year of diagnosis was treated as an ordinal variable and categorized based on the observed quartiles of year of diagnosis (1988-1995, 1996-2001, 2002-2005, and 2006-2009). Information regarding the use of chemotherapy, local control, performance status, and specific radiotherapy technique (i.e., dose, fractionation, beam energy) was not available from the SEER database.

### Outcomes of Interest

Overall survival was the primary study endpoint. Overall survival was defined as the interval from diagnosis to the date of death from any cause. Cause-specific survival was defined as the interval from diagnosis to the date of death from Hodgkin’s lymphoma. Patients who died of causes other than Hodgkin’s lymphoma were censored for the Cause-specific survival analysis. For each subject, a second malignancy was defined as any subsequent malignancy diagnosed following the index diagnosis of NLPHL. Time to second malignancy (latency) was determined as the interval from diagnosis of NLPHL to the date of diagnosis of the second malignancy. Freedom from second malignancy was defined as the proportion of patients without a second malignancy. Each second malignancy was further defined as possibly radiotherapy-related or non-radiotherapy related. Radiotherapy-related second malignancy was defined based on Cahan’s criteria, and was one that occurred in a region of the body that would be irradiated during radiotherapy for NLPHL [11]. A non-radiotherapy-related second malignancy was defined as one that occurred in an area not geographically within standard radiotherapy involved fields. Patients with NLPHL involving multiple nodal regions and those with a second malignancy involving multiple regions were excluded from radiotherapy-related second malignancy analysis because there was not sufficient data on the sites of involvement to identify irradiated regions (identified as patients with unknown radiotherapy-related status).

### Statistical Analysis

The chi-square test and two sample t-test were used to compare groups based on clinical factors. The Kaplan-Meier method was used to estimate cause-specific survival, overall survival, freedom from second malignancy, and freedom from radiotherapy-related second malignancy. Univariate analysis was performed to identify individual covariates associated with these outcomes, and included all covariates listed above. The Log-rank test was used to compare subgroups, and trend test was used for variables with ordinal subgroups (age and year of diagnosis). Multivariable analysis was performed using Cox Proportional Hazards modeling to identify variables that independently predicted overall survival. The proportional hazards assumption was confirmed prior to analysis. The multivariable model included all covariates used in univariate analysis. Due to the limited number of Hodgkin’s lymphoma-related deaths, a multivariable analysis could not be performed for cause-specific survival. The data were analyzed using Stata/MP version 12.1 (Statacorp, College Station, TX). For all statistical tests, two-sided tests were used, and a p-value of <0.05 was considered statistically significant.

### Sensitivity Analyses

In order to confirm the observed effects of age and year in the multivariable model for overall survival were not only due to the categorization schema used, both were subsequently included in a model as continuous variables, and age was also included as a dichotomous variable (<45 vs. ≥45) based on the significance of this cutoff in other series [4]. To ensure that the observed role in multivariable analysis for overall survival of primary site was not dependent on the grouping of the sites into 4 groups, a separate model was created with each primary site as its own category.

## Results

A total of 18,573 patients were identified who were diagnosed with Hodgkin’s lymphoma. Of this cohort, 840 patients had NLPHL (5% of the entire population with Hodgkin’s lymphoma). Of the patients with NLPHL, 469 patients had initial treatment with radiotherapy and were included in this study.

Table **1** depicts patient and tumor characteristics. The median follow-up for the entire cohort was 6 years (range: 0-22 years). One patient had no follow-up after diagnosis of Hodgkin’s lymphoma. Most subjects were 20-44 years of age (54%), and 70% were male. The most common primary sites were the head and neck (36%), the axilla/arm (26%), and multiple regions of involvement (18%). Extranodal involvement (4%) and B-symptoms (7%) were uncommon. Although stage I disease was more common (68%), the proportion of patients with Stage II disease increased over time (21% in 1988-1995 vs. 40% in 2006-2009; p=0.009). There were no temporal trends in age (p=0.10), gender (p=0.53), primary site (p=0.21), extranodal involvement (p=0.86), and presence of B-symptoms (p=0.24). Stage I patients had longer follow-up than stage II patients (median 7 vs. 5 years; p<0.01). However, there was no difference in age distribution (p=0.52), between stage I and II patients. There was a trend towards a higher proportion with B-symptoms amongst stage II patients (10% vs. 6%; p=0.08).

**Table 1:** Patient and tumor characteristics (n=469).

Characteristic	Overall Population (n=469) [range]
**Median Follow-up (years)**	6 [0-22]
**Median Age (years)**	37 [4-88]
**Age (years)**	
≤19	47 (10%)
20-44	251 (54%)
45-59	106 (23%)
≥60	65 (14%)
**Gender**	
Male	326 (70%)
Female	143 (30%)
**Year of Diagnosis**	
1988-1995	90 (19%)
1996-2001	105 (22%)
2002-2005	129 (28%)
2006-2009	145 (31%)
**Primary Site**	
Head and Neck	171 (36%)
Axilla/Arm	122 (26%)
Multiple Regions	84 (18%)
Inguinal/Leg	58 (12%)
Other	34 (7%)
**Stage**	
I	320 (68%)
II	149 (32%)
**Extranodal Involvement**	
No	448 (96%)
Yes	21 (4%)
**B-symptoms**	
No	436 (93%)
Yes	33 (7%)

### Cause-specific Survival

Over the follow-up period, 7 patients died of Hodgkin’s Lymphoma, and the 10-year cause-specific survival was 98%. On univariate analysis, none of the covariates were associated with cause-specific survival (age at diagnosis [p=0.80], gender [p=0.47], primary site [p=0.54], year of diagnosis [p=0.73], extranodal involvement [p=0.25], presence of B-symptoms [p=0.18], and stage [p=0.48]).

### Overall Survival

**Figure 1:**
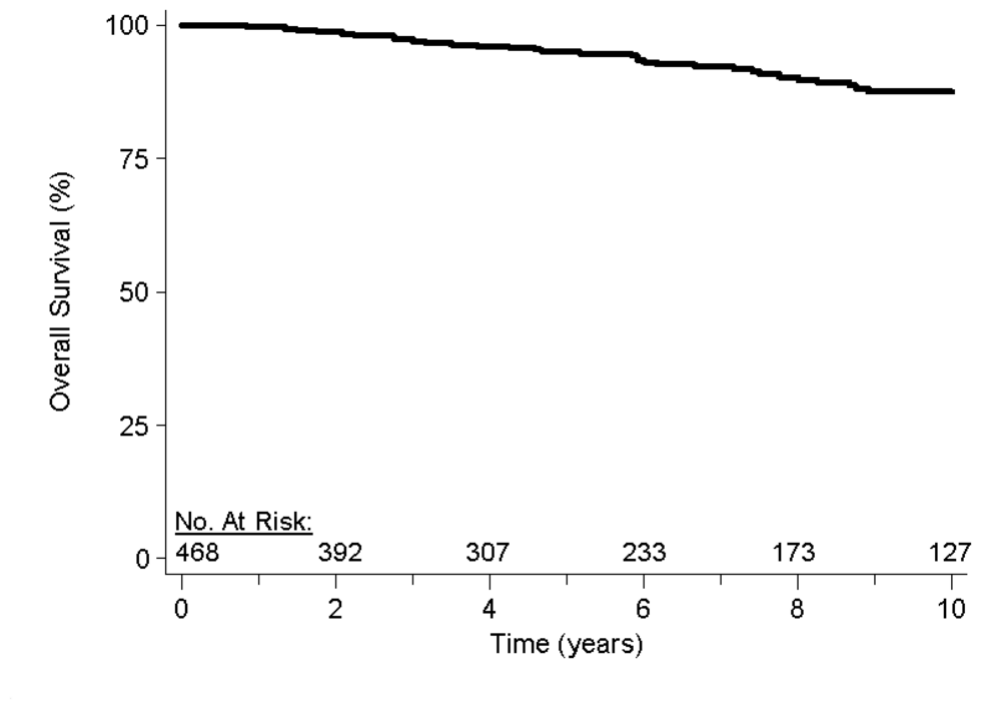
Overall survival. Overall Survival in patients with nodular lymphocyte-predominant Hodgkin’s lymphoma treated with radiotherapy.

Over the follow-up period, 49 patients died. 10-year overall survival for the cohort was 88% (Figure **1**). Table **2** depicts the univariate analysis and multivariable analysis for overall survival. Univariate analysis revealed that increasing age and female gender were associated with worse overall survival. On multivariable analysis, older age, female gender, involvement of multiple regions, and B-symptoms were associated with worse overall survival. Sensitivity analyses confirmed that older age was associated with overall survival when included in the model as a dichotomous variable (<45 vs. ≥45; p<0.01) and as a continuous variable (p<0.01). Additionally, even when each primary site was a category, primary site was not associated with overall survival.

**Table 2:** Univariate and multivariable analysis for overall survival (n=469).

Covariate	10-year Overall Survival	p-value*	Hazard Ratio (95% Confidence Interval)	p-value^†^
**Age (years)**		<0.01^††^		
20-44	95%		Referent	-
≤19	98%		0.63 (0.14-2.88)	0.56
45-59	80%		3.589 (1.73-7.43)	<0.01
≥60	52%		7.942 (3.61-17.49)	<0.01
**Gender**		<0.01		
Male	91%		Referent	-
Female	81%		1.94 (1.03-3.66)	0.04
**Year of Diagnosis**		0.24^††^		
1988-1995	82%		Referent	-
1996-2001	91%		0.56 (0.27-1.19)	0.13
2002-2005	^║^		0.52 (1.00-1.33)	0.17
2006-2009	^¶^		0.47 (0.09-2.04)	0.29
**Primary Site**		0.60		
Head and Neck	90%		Referent	-
Axilla/Arm	88%		1.12 (0.53-2.39)	0.77
Multiple Regions	85%		6.93 (1.22-39.50)	0.03
Inguinal/Leg	89%		1.21 (0.43-3.38)	0.72
Other	80%		0.74 (0.19-2.95)	0.67
**Stage**		0.31		
I	87%		Referent	-
II	90%		0.17 (0.04-0.76)	0.02
**Extranodal Involvement**		0.17		
No	88%		Referent	-
Yes	88%		3.25 (0.73-14.48)	0.12
**B-symptoms**		0.16		
No	88%		Referent	-
Yes	75%		4.47 (1.22-16.37)	0.02

*=Log-rank test

†=Cox Proportional Hazard Modeling

††=Trend test

║=88% alive with 8 years of follow-up

¶=98% alive with 4 years of follow-up

### Second Malignancy

**Figure 2:**
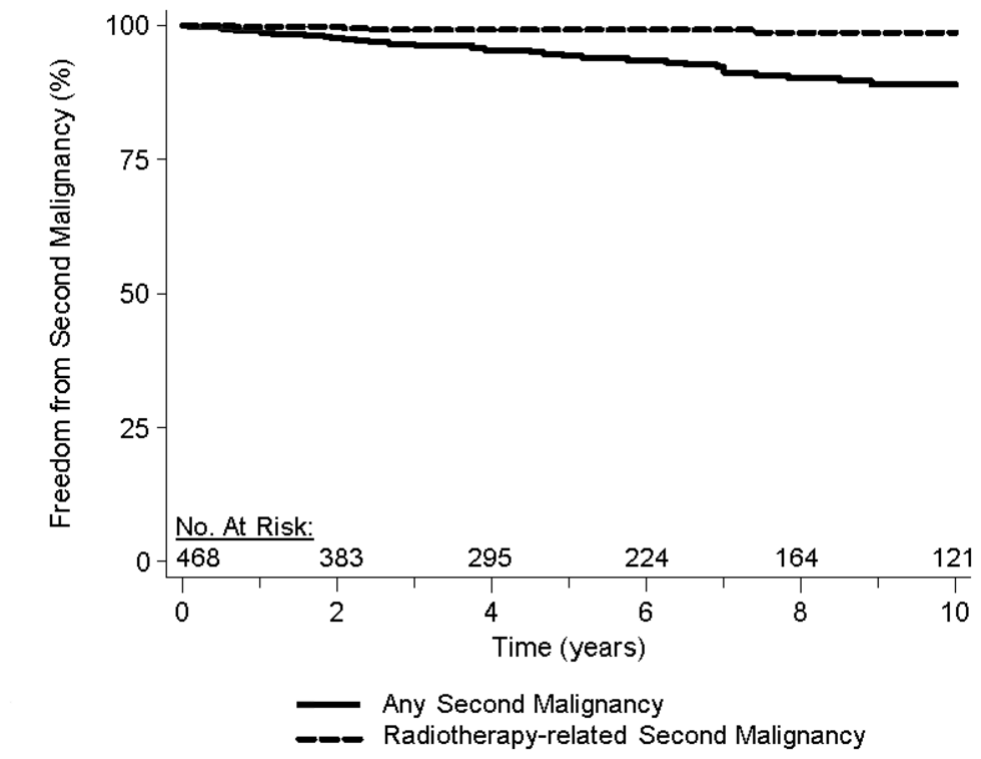
Freedom from any second malignancy and radiotherapy-related second malignancy. Freedom from any second malignancy (solid line) and freedom from radiotherapy-related second malignancy (dashed line) in patients with nodular lymphocyte-predominant Hodgkin’s lymphoma treated with radiotherapy.

Forty-six patients (10% of the overall cohort) developed a second malignancy following NLPHL. Of these, 4 patients subsequently developed a 3^rd^ malignancy (<1% of overall cohort). The median time to second malignancy was 6 years (range: 1 month-15 years). The most common type of second malignancy was non-Hodgkin’s lymphoma (n=13; 28%), while the second most common was lung cancer (n=5; 11%). The 10-year freedom from second malignancy for the entire cohort was 89% (Figure **2**).

Of the patients with a second malignancy, 9 malignancies (20%) were presumed to be radiotherapy-related and 21 were non-radiotherapy-related. In 16 patients, radiotherapy-related status was unknown. Therefore, these patients were excluded from the radiotherapy-related second malignancy analysis. Ten-year freedom from presumed radiotherapy-related second malignancy was 99% for the analyzed cohort (n=453; Figure **2**).

## Discussion

NLPHL is a rare clinical entity with a natural history varying from that of classical Hodgkin’s lymphoma. Prior studies have revealed that patients with early-stage NLPHL can be treated with radiation alone [7,8,9]. The current study evaluated the outcomes of patients with stage I-II NLPHL in a population-based database and found that treatment with radiation therapy results in excellent 10-year cause-specific survival and 10-year overall survival of 98% and 88%, respectively.

A retrospective study from patients treated on the German Hodgkin Study Group HD4-HD12 protocols revealed that a hemoglobin <10.5 g/dl, age ≥45, and advanced stage were all associated with worse overall survival [4]. Our study confirmed the finding that age ≥45 is a negative prognostic factors for overall survival and revealed additional negative prognostic factors including having multiple sites of nodal involvement, female gender, and the presence of B-symptoms. Interestingly, stage I patients had an independently worse overall survival compared to stage II patients despite having no difference in cause-specific survival. However, the absolute difference was small (10-year overall survival of 87% vs. 90%). In the series by Chen et al, stage II patients had a numerically improved 10-year overall survival (97% vs. 94%) compared to stage I patients (97% vs. 94%), but this was not statistically significant (p=0.53) [8]. Another series, by Wirth et al, found that stage did not predict survival in univariate or multivariable analysis [7]. In our series, the worse outcomes of stage I patients was likely due to the larger power to detect small differences and the longer follow-up of stage I patients compared to stage II patients.

Table **3** compares the patient characteristics, overall survival, and second malignancy incidence of the current study to that of the largest published studies in patients with NLPHL [4,5,7,8,9,12]. The 10-year overall survival of patients in our series was similar to most other series in the literature. Patients with classical Hodgkin’s lymphoma are known to have an increased risk of developing second malignancies [13,14,15,16]. We examined this second malignancy risk in the NLPHL cohort and found a 10-year freedom from second malignancy of 89%. The risk of subsequent non-Hodgkin lymphoma in our series was 3%, which is similar to other studies (2-4%) [5,7]. By correlating the initial site of involvement of NLPHL with the location of the second malignancy we were able to surmise whether subsequent second malignancies were radiation induced and found a 10-year freedom from radiotherapy-related second malignancy of 99%. This difference suggests that this patient population has an inherent risk of developing second malignancies regardless of the therapy received because the rate of developing radiotherapy-related second malignancies was so low.

**Table 3:** Recent series evaluating outcomes of patients with nodular lymphocyte-predominant Hodgkin’s lymphoma treated with radiotherapy.

	N	Years	Treatment	Stage I vs. Stage II	B-Symptoms	ENI	Median Follow-up	Overall Survival	Poor Prognostic factors	Incidence of Second Malignancy
International* [5]	219	1980s	>90% RT or CRT	53% vs. 28%	10%	NR	6.8 years	14% died	advanced stage; older age	5% SM
Austrailian [7]	202	1969-1995	100% RT	80% vs. 20%	3%	1%	15 years	83% at 15 years; 88% at 10 years	age ≥45; B-symptoms; number of sites	11% SM; 4% In RT field
Harvard [8]	113	1970-2005	82% RT only; 12% CRT; 6% Chemoonly	63% vs. 37%	NR	NR	11.3 years	Stage I: 94% Stage II: 97%	CRT associated with worse OS; Chemoonly associated with worse PFS	13% SM; 7% in RT field
GHSG* [4]	394	1988-2003	Various Trial Treatments	79% total	9%	6%	4.2 years	Early favorable: 2.4% died; Early unfavorable: 5.3% died	Hb <10.5 g/dl; age ≥45; advanced stage	2.5% SM
Florida* [9]	34	1968-2005	80% RT only; 20% CRT	53% vs. 41%	3%	0%	12.3 years	85% at 10 years; 76% at 15 years	NR	9% SM; 3% in RT field
UK* ^,†^ [12]	42	1982-2000	52% RT	57% vs. 26%	NR	NR	8.9 years	1 death (of disease)	NR	0%
Current Study	469	1988-2009	100% RT^††^	68% vs. 32%	7%	4%	6 years	10-years: 88%	age≥45; multiple involved regions; female gender; B-symptoms	10% SM; 2% RT-related

CRT=chemotherapy and radiotherapy

ENI=extranodal involvement

GHSG=German Hodgkin Study Group

Hb=hemoglobin

NR=not reported

OS=overall survival

PFS=progression-free survival

RT=radiotherapy

SM=second malignancy

*=Included patients with stage III-IV Hodgkin lymphoma

†=children only

††=chemotherapy data not available

This study has the inherent limitations of a retrospective review. One significant limitation is the lack of details regarding radiation therapy. The radiotherapy fields and doses used in the therapy of Hodgkin’s lymphoma have changed over time. Additionally, we do not have information on recurrences or patterns of failure, which are particularly important endpoints due to the favorable survival outcomes in patients with NLPHL. Another limitation is that there was no central pathological review of the initial diagnosis or second malignancy. Histologic diagnosis of NLPHL can be a difficult diagnosis, and thus this could have biased the results [5]. We also did not have information regarding chemotherapy use. However, because only 7% of patients in our cohort would be considered to have stage IB or IIB disease, chemotherapy use in our cohort should have been rare. Additionally, we did not have information on the presence or extent of mediastinal involvement, the erythrocyte-sedimentation rate, or exact number of involved sites. These factors are associated with unfavorable survival in Hodgkin’s lymphoma [17,18,19]. Knowledge of these factors may have allowed for better identification of risk factors associated with survival. Additionally, we cannot make conclusions about late survival outcomes and the incidence of second malignancies at time points later than the duration of follow-up in our cohort. Importantly, only 127 patients had ≥10 years follow-up, suggesting that longer follow-up may be necessary to ascertain the survival and second malignancy outcomes of this cohort of patients. Lastly, we did not have data on any salvage therapy used, which may be of importance due to the favorable survival outcomes of these patients.

In conclusion, this study is the largest series of patients with early-stage NLPHL treated with radiation therapy. Our findings reveal that this patient population has excellent long term overall survival and a low rate of developing radiotherapy-related second malignancy. 
